# Proteome data to explore the impact of pBClin15 on *Bacillus cereus* ATCC 14579

**DOI:** 10.1016/j.dib.2016.07.042

**Published:** 2016-07-26

**Authors:** Jean-Paul Madeira, Béatrice Alpha-Bazin, Jean Armengaud, Hélène Omer, Catherine Duport

**Affiliations:** a SQPOV, UMR0408, Avignon Université, INRA, F-84914 Avignon, France; b CEA, DSV, IBiTec-S, SPI, Li2D,Laboratory "Innovative technologies for Detection and Diagnostics", Bagnols-sur-Cèze F-30200, France

## Abstract

This data article reports changes in the cellular and exoproteome of *B. cereus* cured from pBClin15.Time-course changes of proteins were assessed by high-throughput nanoLC-MS/MS. We report all the peptides and proteins identified and quantified in *B. cereus* with and without pBClin15. Proteins were classified into functional groups using the information available in the KEGG classification and we reported their abundance in term of normalized spectral abundance factor. The repertoire of experimentally confirmed proteins of *B. cereus* presented here is the largest ever reported, and provides new insights into the interplay between pBClin15 and its host *B. cereus* ATCC 14579. The data reported here is related to a published shotgun proteomics analysis regarding the role of pBClin15, “Deciphering the interactions between the *Bacillus cereus* linear plasmid, pBClin15, and its host by high-throughput comparative proteomics” Madeira et al. [1]. All the associated mass spectrometry data have been deposited in the ProteomeXchange Consortium (http://proteomecentral.proteomexchange.org) via the PRIDE partner repository (http://www.ebi.ac.uk/pride/), with the dataset identifier PRIDE: PXD001568, PRIDE: PXD002788 and PRIDE: PXD002789.

**Specifications Table**

**Table:** 

Subject area	*Proteomics*
More specific subject area	*Microbial proteomics*
Type of data	*Figure, Tables*
How data was acquired	*NanoLC-MS/MS using an LTQ-Orbitrap XL hybrid mass spectrometer (ThermoFisher) coupled to an Ultimate 3000 nRSLC system (Dionex, ThermoFisher).*
Data format	*Analyzed*
Experimental factors	*Bacillus cereus cells with and without pBClin15 plasmid*
Experimental features	*Proteins were extracted from bacterial cultures harvested at the early exponential, late exponential and stationary growth phases. The extracellular proteins were obtained by trichloroacetic acid precipitation of culture supernatant. The cellular proteins were obtained after disruption of bacteria with a Precellys 24 disruptor (Bertin Technologies).*
Data source location	*France*
Data accessibility	*Analyzed datasets are within this article and raw data are available via the PRIDE partner repository (*http://www.ebi.ac.uk/pride*) with the dataset identifiers, PRIDE:*PXD001568*, PRIDE:*PXD002788*and PRIDE:*PXD002789.

**Value of the data**

• This is the first proteomic study that assesses the influence of plasmid curation on a bacterium.
• This large proteomic dataset on *B. cereus* is a valuable resource for understanding the relationships between linear plasmids and bacterial cells.
• The data are presented as a reference for other investigators who like to check other functional changes of *B. cereus* whole-cell proteome or exoproteome.

## 1 Data

In this paper we provide the list of the 44 proteins of *B. cereus* that we found previously by proteogenomics (unpublished work) and the lists of proteins identified in the cellular proteome and exoproteome of the ΔpBClin15 and wild-type ATCC 14579 strains [1]. These files comprise label-free quantitation of the proteins based on spectral counts estimated for the three biological replicates in early exponential, late exponential and stationary growth phase. The average log_2_-abundance level for each protein across all samples, *t*-statistics, *p*-values, *p*-value adjusted for multiple testing, and *B*-statistics are provided [2].

## 2 Experimental design, materials and methods

**Fig. 1:**
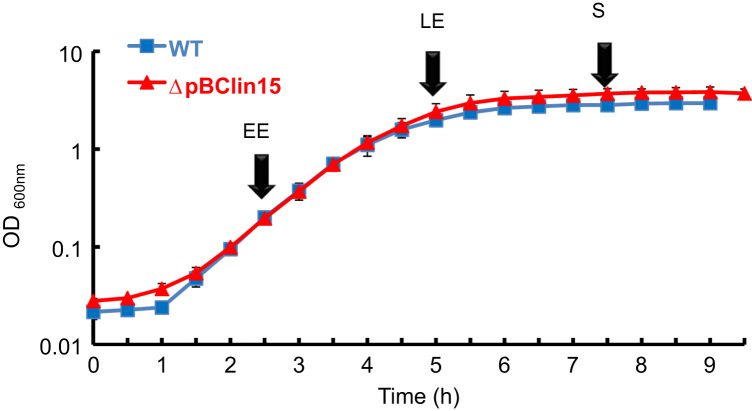
Time course of ΔpBClin15 and its parental strain, ATCC 14,579 (WT). The strains were cultured in MOD medium supplemented with 30 mM glucose under aerobiosis. Samples from ΔpBClin15 and WT were isolated from the early exponential (EE), late exponential (LE) and stationary (S) growth phases as indicated by the arrows.

*B. cereus* cells (with and without pBClin15, [3], [4]) were cultured in batches as described previously [5] with three independent cultures for each strain. Fig. 1 depicts the collection points depending on the time-growth curve, *i.e.* during early exponential growth phase (EE), at the late exponential growth phase (LE) signifying the transition between exponential and stationary phases, and during the stationary phase (S). At each time-point, cells and culture supernatant were collected resulting into two fractions: cellular soluble proteome and exoproteome, respectively.

### 2.1 Sample preparation and shotgun tandem mass spectrometry

Soluble proteins were extracted and processed as described previously [5], [6], [7]. Briefly, total protein samples were loaded onto denaturing NuPAGE 4–12% Bis-Tris gels (Invitrogen) for a 3 mm electrophoretic migration. Proteins were then subjected to proteolysis with sequencing grade trypsin (Roche) using ProteaseMAX surfactant (Promega) at 0.01% [7], [8]. The resulting peptides were analyzed by tandem mass spectrometry as described previously [5], [9]. The peptides from the extracellular digests and those from cellular digests were resolved by reverse chromatography using a 90-min gradient, or a 180-min gradient, respectively, from 4 to 40% solvent B (0.01% HCOOH, 100% CH_3_CN) with solvent A being 0.01% HCOOH, 100% H_2_O.

### 2.2 Database searching and criteria

Tandem mass spectrometry raw data were assigned to peptide sequences with the MASCOT search engine (version 2.3.02) from Matrix Science, the following parameters: full-trypsin specificity, a mass tolerance of 5 ppm on the parent ion and 0.5 Da on the MS/MS, carboxyamidomethylated Cys (+57.0215) as a fixed modification and oxidized methionine (+15.9949) as a variable modification, and a maximum of two missed cleavages. All peptide matches with a score below a *p*-value of 0.05 were filtered by the IRMa 1.28.0 parser [10]. A protein was considered validated when at least two different peptides were identified when considering all the samples. In terms of protein identification, the false-positive rate estimated using the appropriate decoy database resulted below 0.1%.

### 2.3 Data analysis

Spectral counts corresponding to the number of MS/MS spectra per protein were extracted in the three different nanoLC-MS/MS biological replicates for each growth phase. The normalized spectral abundance factor (NSAF) for each protein was calculated by dividing the number of spectral count (SC) by the protein length (L), divided by the sum of SC/L for all N proteins in the experiment [11]. Analyses of abundance level change of proteins were performed with the LIMMA package by the LIMMA Voom method [12]. The data were normalized with the trimmed mean of M-values (TMM) normalization. Then, quantitative proteomics data were analyzed by empirical Bayes moderation of the standard errors towards a common value.
