# Supplementary material for: Proteome data to explore the impact of pBClin15 on Bacillus cereus ATCC 14579
Source: Data Brief. 2016 Jul 26;8:1243–6. doi: 10.1016/j.dib.2016.07.042 (PMC4983103; doi:10.1016/j.dib.2016.07.042)
Supplement: Supplementary file 1 — Supplementary material [file mmc1.docx]

Conflicts of interest : none
